# The impact of first-trimester subchorionic hematomas on pregnancy outcomes after euploid embryo transfer: a retrospective cohort study

**DOI:** 10.1186/s12884-024-06359-5

**Published:** 2024-03-07

**Authors:** Weilin Wang, Qing Zhao, Yingbo Liu, Ling Guo, Wei Zhou, Qian Zhang, Junhao Yan, Tianxiang Ni

**Affiliations:** 1 Center for Reproductive Medicine, Shandong University, Jinan, 250012 Shandong China; 2 Key laboratory of Reproductive Endocrinology of Ministry of Education, Shandong University, Jinan, 250012 Shandong China; 3 Shandong Key Laboratory of Reproductive Medicine, Jinan, 250012 Shandong China; 4 Shandong Provincial Clinical Research Center for Reproductive Health, Jinan, 250012 Shandong China; 5 Shandong Technology Innovation Center for Reproductive Health, Jinan, 250012 Shandong China; 6 National Research Center for Assisted Reproductive Technology and Reproductive Genetics, Shandong University, Jinan, 250012 Shandong China

**Keywords:** Subchorionic hematoma, Preimplantation genetic testing, Pregnancy outcomes, Maternal complications

## Abstract

**Background:**

The aim of the retrospective cohort study was to investigate the prognostic effect of subchorionic hematomas (SCH) in the first trimester on pregnancy outcomes after euploid embryo transfer.

**Methods:**

We retrospectively analyzed women achieving singleton pregnancy by PGT-A or PGT-SR from January 2017 to January 2022. Patients were enrolled in the study if they had a viable intrauterine pregnancy at ultrasound between 6 0/7 and 8 0/7 weeks of gestation. Pregnancy outcomes as well as the incidence of maternal complications were compared between patients with and without SCH. Logistic regression was used for adjusting for potential confounding factors.

**Results:**

A total of 1539 women were included, of which 298 with SCH and 1241 with non-SCH. The early miscarriage rate in SCH group was significantly higher than that in the non-SCH group (10.1% vs. 5.6%, adjusted odds ratio [aOR] 1.99, 95% confidence interval [CI] 1.25–3.16, *P* = 0.003). The live birth rate in SCH group was significantly lower than that in the non-SCH group. (85.6% vs. 91.2%, aOR 0.57, 95% CI 0.39–0.84, *P* = 0.005). In addition, SCH group had an increased risk of hypertensive disorder of pregnancy (HDP) (8.9% vs. 5.2%, *P* = 0.022), especially in hematoma with bleeding (19.3% vs. 6.0%, *P* = 0.002). The incidence of gestational diabetes mellitus (GDM), major congenital abnormalities rate, normal birth weight rate and low birth weight rate were similar between the two groups.

**Conclusions:**

The presence of SCH in the first trimester was associated with worse pregnancy outcomes after euploid embryo transfer, including an increased risk of early miscarriage and hypertensive disorder of pregnancy, along with a reduced live birth rate.

## Introduction

Subchorionic hematoma (SCH) is one of the most common and recognizable abnormalities in first-trimester pregnancy and it is defined as fluid collection between the uterine wall and gestational sac observed through ultrasound scans [1]. The reported incidence of SCH varies widely, from as low as 0.46% to as high as 39.5% [2–4] and it seems to be increased during pregnancy achieved via assisted reproductive technology compared with spontaneous pregnancy [5].

The reported conclusions regarding prognostic impact of SCH on pregnancy outcomes remain conflicting. A large meta-analysis has demonstrated that SCH may increase the risk of pregnancy loss [6], and some studies have found an association between SCH and a higher risk of stillbirth, preterm birth [7] and low birth weight [8, 9], while other studies have reported negative evidence [8, 10, 11]. It has also been documented that SCH characteristic, such as timing, size and presence of vaginal bleeding may affect rates of pregnancy loss and ongoing pregnancies [4, 9, 12].

Above studies only explore the correlation of SCH with pregnancy and perinatal outcomes in the general spontaneous-conceived or IVF/ICSI population. None of these studies can rule out the important confounding factor of the embryo’s own chromosomal abnormalities. In recent years, preimplantation genetic testing (PGT) technology, as a detection technology for the analysis of chromosomal abnormalities in embryos, has been increasingly used worldwide to improve the pregnancy outcomes of people with a poor prognosis [13]. However, since trophectoderm biopsy removes cells destined to form the placenta, the biopsy procedure may have adverse effects on initial placental function and pregnancy and perinatal outcomes. Given SCH as placenta-related bleeding [14], it is unclear whether SCH has adverse effects on pregnancy outcomes after euploid embryo transfer in the context of PGT. Therefore, this study aims to investigate the association between SCH in the first trimester and pregnancy outcomes, as well as the incidence of maternal complications in women achieving singleton pregnancies after euploid embryo transfer.

## Materials and methods

### Patients

This was a retrospective cohort study among women with PGT-A or PGT-SR treatment in a single center for reproductive medicine from January 2017 to January 2022. Patients were enrolled in the study if they had a viable intrauterine pregnancy at ultrasound between 6 0/7and 8 0/7 weeks of gestation. If the patients had multiple transfer cycles obtaining intrauterine pregnancies during this period, only the data of the first embryo transfer was included in this study. In our center, ultrasound examination was performed routinely for every patient during the period of 6 0/7 to 8 0/7 weeks of gestation to determine if a clinical pregnancy was achieved and followed by ultrasound examination on 11 0/7 and 13 0/7 weeks to evaluate ongoing pregnancy and fetal growth. Subchorionic hematoma was defined as a crescent-shaped collection of fluid between the chorionic membrane and the uterine wall [1]. All scans included a standardized check of SCH according to the definition, which included a “yes or no” determination. If SCH was visible, the ultrasound technician would measure its size. The maximum diameter of the length, width and height of the hematoma was displayed through longitudinal and transverse image [1] and the SCH size was represented by the ratio of SCH volume to gestational sac volume [12]. We reviewed the data from each ultrasound report to identify the presence of SCH in the first trimester and recorded the characteristics of SCH, such as size, number. Exclusion criteria included the use of donated oocytes or sperm to achieve pregnancy, twin pregnancies, intentional abortion, ectopic pregnancies, a plan to undergo preimplantation genetic testing for monogenic disease and follow-up loss.

This study has received ethical approval from the Instiutional Review Board of Reproductive Medicine from the Reproductive Center of Shandong Universtiy for the use and analyses of the anonymous data from the patients.

### PGT procedures

The PGT process involved performing a controlled ovarian hyperstimulation (COH), followed by embryo culture in vitro and preimplantation genetic testing. Based on the ovarian reserve, such as age [15], anti-Mullerian hormone (AMH) levels and antral follicle count (AFC) [16], as well as previous ovarian response, several conventional gonadotropin regimens, including long and short gonadotropin-releasing hormone (GnRH) agonists, super-long GnRH agonists, antagonists, and mild stimulation protocols, were used. When at least two follicles ≥ 1.8 cm in diameter were detected, final oocyte maturation was triggered by human choionic gonadotophin (hCG) and transvaginal ultrasound-guided oocyte retrieval was performed 34-36 h after trigger injection. Fertilization was all achieved through intracytoplasmic sperm injection (ICSI). Trophectoderm biopsy was performed on good-quality blastocysts selected by Gardner criteria [17] on Day 5 or Day 6 of embryo culture. Biopsied samples were genetically analyzed using next generation sequencing. Only single embryo transfer was allowed. Endometrial preparation mainly included natural ovulation cycle, artificial regimen and ovulation induction cycle. The natural ovulation cycle was mainly suitable for women with regular menstrual cycles and normal ovulation. To patients with oligomenorrhea or irregular menstruation, such as polycystic ovary syndrome, premature ovarian failure, endometriosis, artificial regimen was recommended. If the patient had poor endometrial outcome after the natural ovulation cycle or artificial regimen, an ovulation induction cycle or other methods may be used. Luteal support was given when endometrial thickness reached ≥ 7 mm and continued until 12 weeks of gestation. The pregnancy outcomes and maternal complications were followed up and recorded in detail.

### Outcome measures

The primary outcome in this study was the rates of miscarriage and live birth. Miscarriage was defined as cessation of pregnancy prior to 28 weeks. Among them, cessation of pregnancy before 12 weeks was considered to be early miscarriage, more than 12 weeks but prior to 28 weeks was considered to be late miscarriage. Live birth was defined as the birth of any viable newborn who was 28 weeks of gestation or older [18].

The second outcomes included the rate of preterm birth, the incidence of major congenital abnormalities and birth weight of newborns. Preterm birth was defined as the delivery before 37 completed weeks of gestational age. Normal birth weight was defined as the birth weight of newborn between 2500 gram to 4000 gram and low birth weight meant less than 2500 gram.

Other outcomes included the incidence of maternal complications, particularly hypertensive disorder of pregnancy (HDP) and gestational diabetes mellitus (GDM).

### Statistical analysis

Continuous variables were described as mean ± SD or median (interquartile range). Categoric variables were represented as frequency and percentage. Student t test was used to compare normally distributed continuous variables and Mann-Whitney U test was used for nonparametric variables. Pearson’s chi-square test or Fisher’s exact test were used for categorical variables. Logistic regression analysis was used to determine the associations between the presence of SCH and miscarriage or live birth after adjusting for the following cofounders: maternal age, body mass index (BMI), gonadotropin total dose, basal estrogen levels, luteinizing hormone, endometrial thickness on transfer day and vaginal bleeding. A probability (P) value of < 0.05 was considered statistically significant. All statistical analyses were performed by IBM SPSS26.0 software.

## Results

### Baseline characteristics

A total of 1539 pregnancies were included in this study, of which 298 were diagnosed with SCH and 1241 were not. The prevalence of SCH in the first trimester was 19.3%.

Baseline characteristics of the two groups were demonstrated in Table 1. Patients with SCH were more likely to have a higher level of basal E2 (36.5 vs. 34.8, *P* = 0.027) and were more likely to have vaginal bleeding (22.5% vs. 11.6%, *P*<0.001). There were no differences in other clinical baseline characteristics between the two groups (Table 1). Similarly, there were also no difference in the results of controlled ovarian hyperstimulation and embryo culture between the two groups (Table 2).

**Table 1:** Baseline characteristics for groups with and without subchorionic hematoma (SCH)

Variable	SCH (*n* = 298)	Non-SCH (*n* = 1241)	P-value
Maternal age (y)	32.3 ± 4.7	32.2 ± 4.8	0.561
BMI (kg/m2)	23.5 ± 3.3	23.8 ± 3.4	0.070
AMH (ng/mL)	3.5 (1.8, 6.1) (290) ^a^	3.6 (2.0, 6.0) (1220) ^a^	0.497
chromosomal abnormalities for PGT-SR	107(35.9)	497(40.0)	0.189
No. of Previous miscarriage			0.776
0	88 (29.5)	388 (31.3)	
1	67 (22.5)	272 (21.9)	
2	72 (24.2)	269 (21.7)	
≥ 3	71 (23.8)	312 (25.1)	
Basal hormone levels^b^			
Follicle-stimulating hormone (IU/liter)	6.5 ± 1.9	6.4 ± 1.9 (1239) ^a^	0.746
Luteinizing hormone (IU/liter)	6.1 ± 4.6	5.6 ± 3.6 (1239) ^a^	0.140
Estradiol (pg/ml)	36.5 (27.4, 49.2) (297) ^a^	34.8 (26.8, 44.4) (1229) ^a^	0.027
Ultrasonographic findings^c^			
AFC	16.9 ± 8.8	16.4 ± 8.2 (1236) ^a^	0.338
PCO	81 (27.2)	326 (26.3)	0.749
Uterine fibroids	51 (17.1)	217 (17.5)	0.879
Vaginal bleeding	67 (22.5)	144 (11.6)	<0.001

Data are presented as mean ± standard deviation or median (interquartile range) or n (%) unless otherwise specified. AFC, antral follicle count; AMH, anti-Mullerian hormone; BMI, body mass index; PCO, polycystic ovaries; SCH, subchorionic hematoma

^a^Number in brackets represents the number of observations that was less than the total patient number in the group

^b^The baseline steroid hormones were measured at the early follicular phase, mostly on day 1 to 3 of the menstrual cycle

^c^Ultrasound is performed at the early follicular phase, mainly on days 1–3 of the menstrual cycle

**Table 2:** Results of controlled ovarian hyperstimulation, embryo culture and transfer

Variable	SCH (*n* = 298)	Non-SCH (*n* = 1241)	P-value
Ovarian stimulation protocols			0.985
Long	132 (44.3)	561 (45.2)	
Short	51 (17.1)	212 (17.1)	
Antagonist	96 (32.2)	395 (31.8)	
Others^b^	19 (6.4)	73 (5.9)	
Gonadotropin total dose	1843.5 ± 813.9	1944.8 ± 882.7	0.071
No. of oocytes obtained	13.4 ± 6.6	14.0 ± 7.3	0.167
No. of MII oocytes	11.9 ± 6.0	12.2 ± 6.2	0.458
Rate of MII oocytes	0.9 (0.8, 1.0)	0.9 (0.8, 1.0)	0.474
No. of 2PN oocytes	9.3 ± 4.9	9.7 ± 5.0	0.241
Rate of 2PN oocytes	0.7 (0.6, 0.8)	0.7 (0.6, 0.8)	0.659
No. of Good-quality blastocysts	4.0 (3.0, 7.0)	4.0 (3.0, 7.0)	0.494
FET protocol			0.198
NC	155 (52.0)	581 (46.8)	
HRT	100 (33.6)	438 (35.3)	
Others^c^	43 (14.4)	222 (17.9)	
Endometrial thickness on transformation day (cm)^d^	0.9 (0.8, 1.0)	0.9 (0.8, 1.0) (1239)^a^	0.158

Data are presented as mean ± standard deviation or median (interquartile range) or n (%) unless otherwise specified. FET, frozen embryo transfer; PGT-A, preimplantation genetic testing for aneuploidy; PGT-SR, preimplantation genetic testing for chromosome structural rearrangements; SCH, subchorionic hematoma; NC, natural cycle; HRT, hormone replacement treatment

^a^Number in brackets represents the number of observations that was less than the total patient number in the group

^b^Others include minimal ovarian stimulation protocol and super-long protocol

^c^Others include modified natural cycle, ovulation induction cycle and down-regulation plus HRT cycle

^d^Endometrial thickness on transformation day means the last measurement of the endometrial thickness before starting progesterone and is usually performed on the day progesterone is used

### Pregnancy outcomes and maternal complications

The pregnancy outcomes were shown in Table 3. Pregnancies with SCH in the first trimester were found to have a significantly increased risk of miscarriage (14.1% vs. 8.5%, *P* = 0.003) and a reduced rate of live birth (85.6% vs. 91.2%, *P* = 0.003). When analyzed separately according to the gestational age of miscarriage, there was a significantly higher risk of early miscarriage (10.1% vs. 5.6%, *P* = 0.004) whereas no difference in the rate of late miscarriage between the two groups (4.0% vs. 2.9%, *P* = 0.315). After adjusting for confounders, the presence of SCH still significantly correlated with an elevated risk of early miscarriage and a decreased live birth rate (aOR for early miscarriage 1.99; 95% confidence index [CI], 1.25–3.16; *P* = 0.003; aOR for live birth 0.57; 95% CI, 0.39–0.84; *P* = 0.005) (Table 4). Preterm birth rate, the incidence of major congenital abnormalities and low birth weight were similar between groups.

**Table 3:** Pregnancy outcomes as well as the maternal complications for groups with and without SCH

Variable	SCH (*n* = 298)	Non-SCH (*n* = 1241)	P-value
Miscarriage rate	42 (14.1)	105 (8.5)	0.003
Early miscarriage rate	30 (10.1)	69 (5.6)	0.004
Late miscarriage rate	12 (4.0)	36 (2.9)	0.315
Live birth rate	255 (85.6)	1132 (91.2)	0.003
Preterm delivery rate	18 (6.0)	81 (6.5)	0.758
Normal birth weight rate	223 (87.5)	977 (86.6)	0.721
Low birth weight rate	10 (3.9)	45 (4.0)	0.960
Major congenital abnormalities rate	7 (2.7)	49 (4.3)	0.246
HDP	23 (8.9)	59 (5.2)	0.022
Eclampsia and preeclampsia	3 (1.2)	13 (1.1)	0.979
GDM	39 (15.1)	148 (13.0)	0.372

Data are presented as mean ± standard deviation or median (interquartile range) or n (%) unless otherwise specified. GDM, gestational diabetes mellitus; HDP, hypertension disorders of pregnancy

**Table 4:** Adjusted odds ratio of predictive variables and participants with primary pregnancy outcomes

	Early miscarriage		Live birth	
Variable	aOR (95% CI)	P-value	aOR (95% CI)	P-value
SCH		0.003		0.005
No	1		1	
Yes	1.99 (1.25–3.16)		0.57 (0.39–0.84)	
Maternal age (y)	1.05 (1.01–1.10)	0.021	0.95 (0.91–0.98)	0.003
BMI	1.08 (1.01–1.15)	0.017	0.92 (0.88–0.97)	0.003
Gonadotropin total dose	1.00 (1.00–1.00)	0.195	1.00 (1.00–1.00)	0.247
Estradiol (pg/ml)	1.00 (1.00–1.00)	0.969	1.00 (1.00–1.00)	0.922
Luteinizing hormone (IU/liter)	1.01 (0.95–1.06)	0.855	1.00 (0.95–1.04)	0.816
Endometrial thickness on transformation day (cm)	0.11 (0.03–0.43)	0.001	3.46 (1.19–10.07)	0.023
Vaginal bleeding		0.886		0.159
No	1		1	
Yes	1.04 (0.60–1.83)		0.73 (0.47–1.13)	

The covariables in the regression models were maternal age(y), body mass index (BMI), gonadotropin total dose, basal estradiol (pg/ml), luteinizing hormone (IU/liter), endometrial thickness on transfer day(cm) and vaginal bleeding. CI, confidence interval; BMI, body mass index; OR, odds ratio; SCH, subchorionic hematoma

As shown in Table 3, HDP were significantly more common in the SCH group (8.9% vs. 5.2%, *P* = 0.022). However, the proportion of cases that progressed to preeclampsia or even eclampsia did not differ significantly between the two groups. In addition, the incidence of GDM was assessed, but no difference was found.

### Subgroup analyses according to different SCH features

Pregnancy outcomes and maternal complications in groups with different SCH features were presented in Table 5. Subgroup analyses showed that different sizes and quantities of SCH had no significant effect on adverse pregnancy outcomes. Vaginal bleeding, a common clinical symptom of early pregnancy, was also included in the study. We found that SCH patients with bleeding were more likely to develop HDP (19.3% vs. 6.0%, *P* = 0.002).

**Table 5:** Pregnancy outcomes for groups with different SCH features

SCH Features	Miscarriage	Early miscarriage	Live birth	HDP	GDM
Size (%)^a^					
≤ 10%	9 (9.8)	6 (6.5)	84 (90.3)	7 (8.4)	12 (14.5)
11–25%	8 (11.4)	7 (10.0)	64 (87.7)	5 (7.9)	12 (15.9)
26–50%	12 (17.9)	8 (11.9)	57 (81.4)	2 (3.6)	11 (18.2)
>50%	13 (22.8)	9 (15.8)	46 (79.3)	9 (20.0)	5 (11.1)
P-value	0.116	0.332	0.194	0.054	0.795
No. of SCH					
1	38 (13.5)	26 (9.2)	244 (86.5)	22 (8.9)	37 (15.0)
≥ 2	4 (25.0)	4 (25.0)	11 (68.8)	1 (8.3)	2 (16.7)
P-value	0.358	0.107	0.109	>0.990	>0.990
Vaginal bleeding					
No	33 (14.2)	25 (10.8)	198 (85.3)	12 (6.0)	30 (14.5)
Yes	9 (13.6)	5 (7.6)	57 (86.4)	11 (19.3)	12 (20.3)
P-value	0.904	0.446	0.835	0.002	0.318

Data are presented as n (%) unless otherwise specified. SCH, subchorionic hematoma; GDM, gestational diabetes mellitus; HDP, hypertension disorders of pregnancy

^a^The size of SCH represent volume (length*width*height) of the hematoma estimated as a fraction of gestational sac volume (length*width*height)

## Discussion

This is the first study to report the association between the presence of SCH in the first trimester and the pregnancy outcomes among euploid embryo transfer population. We found that SCH during early pregnancy was associated with an increased risk of early miscarriage and a reduced live birth rate, regardless of the occurrence of vaginal bleeding. We also found that women with SCH had an increased risk of HDP. Whereas, we found no evidence of elevated risk of preterm birth, GDM, and major congenital abnormalities in women with SCH.

SCH occurred in 19.3% of pregnancies in our cohort, which approached the upper limit of the reporting range of spontaneous pregnancies (0.5-22%), but was consistent with the study among women with spontaneous pregnancy in 2019 [10, 11] and the study among IVF women in 2020 [1]. There were two possible reasons account for the slightly higher SCH incidence in this study. First, ultrasound examination was performed more frequently (at least two times) in this study, which was the routine clinical procedure of PGT-A or PGT-SR treatment, while patients with spontaneous pregnancy usually performed ultrasound to detect SCH when vaginal bleeding or other symptoms occurred. In addition, it has been suggested by some studies that there was a higher incidence of complications associated with placental abnormalities in assisted reproductive technology pregnancies, especially PGT treatment [19], and that SCH may be the result of blood vessel rupture during abnormal invasion of villi into the endometrium [20, 21].

Our findings were consistent with the conclusion of a recent large meta-analysis that reported the positive correlation of SCH with early miscarriage, and no correlation with preterm birth [6]. However, there were conflicting conclusions from other studies evaluating the prognostic impact of SCH on pregnancy outcomes. Two studies in 2019 showed that SCH was not associated with pregnancy loss before 20 weeks of gestation or adverse outcomes after 20 weeks of gestation in the natural pregnancy cohort [10, 11]. For IVF population, a study in 2020 reported that SCH in early pregnancy was not associated with the probability of live birth, preterm birth, or infant birth weight [1]. In contrast, a study in 2017 found that the birth weight of SCH singleton pregnancies in IVF/ICSI was lower than that of SCH-free singleton pregnancies, but also not associated with pregnancy loss [8]. For infertile individuals, previous study reported no increased risk of miscarriage [22]. However, when patients noticed both vaginal bleeding and cramping, the likelihood of miscarriage increased significantly. Previous studies targeted a variety of populations, such as natural pregnancy or IVF/ICSI or both, but cannot avoid the limitation of a significant confounder of aneuploidy in the embryo itself, whereas our study was novel in that it only focused on patients who underwent “euploid” embryo transfer.

The underlying mechanism by which SCH caused early miscarriage in this study mainly contains two aspects. One possible mechanism was premature perfusion of the villous cavity, such as SCH, which occurred before the placenta adapted to oxidative stress, leading to an imbalance between oxidative stress outburst and antioxidant defense [23]. Another possible mechanism was the subchorionic hemorrhage and the secondary mechanical action of the hematoma. Inadequate entry of trophoblast cells into the uterus may impair angiogenesis, and the resulting weak blood vessels may lead to subchorionic hematoma, with adverse consequences [24]. Furthermore, the embryos transferred in this study were all subject to biopsy by removing several trophoblast ectoderm cells, which may impair the antioxidant capacity and recover ability of trophoblast cells after SCH formation, leading to early miscarriage for poor-prognosis patients. In contrast, in both natural and IVF pregnancies, the embryos were intact and the ability of reattachment to the endometrium as the hematoma absorbed may be sufficient for the pregnancy to proceed further. Therefore, the abortion time of PGT-A or PGT-SR patients mainly focused in the first trimester.

In addition, we found that SCH in the first trimester could identify the risk of pregnancy complications. Our study showed that the presence of SCH was associated with an increased risk of hypertension disorders of pregnancy. These diseases were known to be associated with placental defects and invasive cytotrophoblast apoptosis [25]. However, it worth noting that there was no significant difference between the two groups in the incidence of pre-eclampsia and eclampsia, suggesting that although SCH may increase the risk of placental abnormalities, the effects on fetuses and pregnant women may be limited.

Many studies have attempted to correlate SCH characteristics with pregnancy outcomes and determine whether these factors are predictive [26], but the conclusions remained controversial. The effect of SCH size on pregnancy outcomes varied from study to study. This may be due to the different methods of measuring hematoma and the arbitrary definition of hematoma “size” in different studies. A study indicated that the size of the hematoma estimated as a fraction of gestational sac size significantly correlated with first-trimester pregnancy loss [12]. In our study, similar to the conclusion of studies in 2019 [10] and in 2022 [27], the estimated size of SCH was not associated with miscarriage. The presence or absence of a hematoma may be one of the indicators that the placenta was functioning properly, independent of its number and size. Vaginal bleeding, the most common clinical manifestation of subchorionic hematoma, was also considered a predictor of pregnancy loss associated with SCH. Many studies have shown that the presence or absence of vaginal bleeding was not associated with adverse pregnancy outcomes [10, 12]. Nevertheless, we found that SCH with vaginal bleeding was independently associated with the development of HDP. This conclusion needs to be verified with a larger sample size.

One of the strengths of this study was that all embryos transferred were euploid, which avoided chromosomal abnormalities, an important factor contributing to miscarriage. The other one was adequate sample size and the detailed clinical information made good comparability between groups. Our study was limited by retrospective design. Some other placenta-related complications, such as placental abruption, placenta accreta, etc., were not analyzed as we did not have adequate data to support these analyses. In addition, our study only observed SCH at the time of ultrasound examination, duration and change of SCH size during pregnancy may also be important factors in predicting pregnancy outcomes. Therefore, the relationship between hematoma and adverse pregnancy outcomes such as miscarriage after euploid embryo transfer need to be further studied.

## Conclusion

Our study found that the presence of SCH in the first trimester was associated with worse pregnancy outcomes after euploid embryo transfer, including an increased risk of early miscarriage and gestational hypertension, along with a reduced live birth rate. This finding would provide important information for clinicians when SCH is observed and intense surveillance should be given for these patients especially who would have high expectations after transfer of euploid embryos.

## Data Availability

The datasets used and/or analyzed during the current study are available from the corresponding author on reasonable request.
